# Zeolite-amended peat carriers improve long-term survival of *Ensifer meliloti* and maintain alfalfa nodulation efficiency

**DOI:** 10.3934/microbiol.2026029

**Published:** 2026-09-15

**Authors:** Olivera Stajković-Srbinović, Mila Pešić, Biljana Sikirić, Vesna Mrvić, Snežana Andjelković, Dušica Delić

**Affiliations:** 1 Institute of Soil Science, Teodora Drajzera 7, 11000 Belgrade, Serbia; 2 Institute for Forage Crops Kruševac, Globoder, Kruševac, Serbia

**Keywords:** rhizobia, inoculant, peat, nanozeolite

## Abstract

Microbiological N fertilizers containing native highly effective and competitive rhizobial bacteria placed in a suitable carrier are the best choice for sustainable plant production. This study evaluated the ability of various peat-based formulations with different zeolite/CaCO_3_ amendments to support the survival and preservation of symbiotic efficiency of alfalfa rhizobia. 15 different inoculants were tested, including five nitrogen-fixing efficient *Ensifer meliloti* strains (218, 225, 252, 4148ss and 4193cs) in three different carriers made of peat and perlite and supplemented with micro-zeolite (B1), nanozeolite (B2), and micro-zeolite + CaCO_3_ (B3). The number of rhizobia after 90 days of storage at room temperature was above 10^10^ CFU/g of inoculant in most of the combinations, after 180 days of storage was 10^9^ CFU/g in all combinations, and in 330^th^ day stayed above 10^8^ CFU/g of inoculant for strains 218, 4148ss, and 252. Considering the whole period of storage, the most performant carriers were B3, with the addition of micro-zeolite + CaCO_3_ and B2 with nanozeolite amendment. The efficiency of all inoculants, measured by the nodulation ability and shoot dry weight (SDW), was confirmed in inoculated alfalfa plants after six and eleven months of storage. The SDW of inoculated alfalfa plants increased from 2.5 up to 5 times compared to non-inoculated plants, while the nitrogen content (mg/plant) was also significantly increased. The SDW was influenced by strain, while the type of carrier and storage of inoculant did not have an effect. The inoculant formulations with zeolite and calcium carbonate showed good potential for rhizobia survival and effectiveness preservation during long-term storage, which should be further tested under field conditions.

## 1. Introduction

The mineral nitrogen (N) fertilizers are extensively used worldwide with questionable economic viability and environmental pollution effect since the N percentage used from mineral fertilizers by crops is 50%, while the rest N of applied fertilizer is lost by various processes, including leaching and volatilization [1].

Rhizobial inoculants (i.e., microbial N fertilizers) are an alternative [2] or supplement to mineral N fertilizers [3]. Rhizobial bacteria (the most common genera *Rhizobium*, *Ensifer* (*Sinorhizobium*), and *Bradyrhizobium*) are facultative microsymbionts that live independently in the soil, and they can establish a symbiosis with specific legumes by forming nodules on the leguminous root and perform symbiotic nitrogen fixation, which is of considerable environmental and agricultural importance being responsible for most of the atmospheric N fixed in terrestrial ecosystems [4].

Rhizobial inoculants contain selected highly effective and highly competitive rhizobial bacteria placed in suitable carrier. The role of carrier is to maintain the viability and efficiency of bacteria and to increase the shelf life of the inoculant. The formulation of the inoculant involves introducing of the rhizobial bacteria (active agent) into a suitable carrier [5]–[7]. The most widespread formulation consists of peat as the rhizobia carrier [8]. Peat has been used as the carrier in inoculants for decades, mainly because of low cost, easy access, simple production, suitability for many bacterial species, preserving the rhizobia effectiveness in nitrogen fixation [5],[9]–[13]. Generally, peat maintains the viability of rhizobial strains for up to six months of storage, facilitating their manipulation and adhesion to seeds. Other formulation of inoculants compared to peat are sensitive to temperature increases, which reduces the microbial population, the shelf life of liquid inoculants is shorter, and the storage room temperature is less suitable [14],[15]. Therefore, there are constant efforts to improve the peat formulations, particularly for the duration under uncontrolled conditions of storage, as well as the preservation of not only number but also the activity and effectiveness of inoculants.

The inoculant should secure a minimum of about 100 to 20,000 viable bacteria per inoculated seed, depending on the legume, or more to secure optimal nodulation under poor ecological conditions [5],[9],[16]–[18]. This number will be provided with optimal suspension of rhizobia of 10^9^ CFU/g of peat inoculum/inoculant. According to the national regulation, this number has to be a minimum of 10^8^ CFU/g (mL) for symbiotic N-fixers and 10^7^ CFU/g (mL) for plant growth promoting rhizobacteria [19].

The enrichment of the peat carrier by different additives should help to retain moisture, keep a suitable inoculant pH and nutrient of the carrier in the inoculant before the inoculation, but also in the layer of the carrier and surrounding soil on the inoculated seeds, and protect the coated seeds from desiccation [11],[20].

Among other carrier materials for bacterial inoculant, zeolites have been recognized for their potential as carriers or/and additives [21]–[28]. Zeolites have a high water-holding capacity, high cation-exchange capacity, and high adsorption capacity [29]. Moreover, zeolite is a successful carrier for plant growth-promoting rhizobacteria (PGPR) inoculation because it provides a favorable environment to the inoculated bacteria by providing protection and preservation of the bacterial communities, and by creating conditions for expression of their metabolic activities [29]. It is considered to be non-toxic to bacteria [29],[30]. The most common natural form of zeolite is clinoptilolite, which can have the role of an additive because of its characteristics. The ratio Si/Al is greater than 4, which means that clinoptilolite is more hydrophobic than other zeolites, with a greater capacity to absorb materials from aqueous solutions [31]. Zeolites' structures are consisted to have many “channels and cages” that make the crystal-line frameworks accessible to the external substances [32].

Peat and standard seed coats can become locally acidic, thereby inhibiting rhizobial viability so calcium carbonate acts as a stable buffering agent that maintains a neutral-to-mildly alkaline microenvironment optimal for rhizobia [10]. Calcium based additives may improve the inoculum structure, reduce substrate toxicity, and provide essential nutrients (calcium) to microorganisms. Therefore, calcium salts were used in seed priming and peat inoculants [33]–[36].

The recent trend in agriculture for the carrier is the application of an additive in nanogranulation to secure the bacterial environment and maintain bacterial number on seed surface [21],[22]. In comparison with conventional surfaces, nanoparticles have higher surface tension, which has an influence on their potential for absorption and the slow release of the nutrients and moisture from and to the environment. Nanomaterials are characterized by their small size, high surface-to-volume ratio, and unique optical properties. The dimensions of nanoparticles are 100 nm or less. They have a higher reactivity in comparison to the particle size (micron or millimeter size) present in bulk [22],[23]. Nanogranulation of the clinoptilolite by increasing its surface would enhance its water and nutrients retention efficiency in the layer of the inoculant on inoculated seeds. The carriers with smaller particle sizes have a higher surface area, thereby increasing the resistance of bacteria to desiccation by increasing the coverage of bacterial cells; hence, nano-carriers would have superiority over other carriers in this regard [37]. However, a very small substrate particle may result in substrate agglomeration, which can affect the respiration of microorganisms or aeration with a negative result to microbial life, while larger particles can provide a normal respiration efficiency (owing to increased interparticle space) [38]. However, there is still limited data about the interaction of nanozeolite with bacteria, its effects on the survival and functionality of bacteria, as well as the suitability of nanozeolite application in various biological environments.

Supplementing the peat with nanozeolite is a novel attempt in producing more effective peat biofertilizers for small seed legumes such as alfalfa (*Medicago sativa* L.). Therefore, we evaluated the peat and perlite combinations with micronized zeolite, nanozeolite, and CaCO_3_ as carriers for five different *Ensifer meliloti* strains effective in N fixation with alfalfa. The research aims to evaluate whether nano-carrier could enhance the longevity and the bacterial efficiency in N fixation, and to identify the best carrier formulation that could provide long term storage (up to one year) under common conditions of storage (room temperature) and retain the inoculant efficiency, that is, nodulation and plant growth promoting ability in alfalfa.

## 2. Materials and methods

### 2.1. Inoculant preparation

Mineralized peat of pH 7.50 and organic matter content >50% was used in this study as the main carrier to produce the inoculant for alfalfa and was commercially obtained by local supplier. Before use, the peat was dried to 7%–8% moisture, ground, and sieved to a 0.02–0.04 mm particle size [39]. Prepared this way, it was mixed with other materials at different weight ratios. The following solid carrier formulations were examined:

$$\begin{array}{l} \text{B1}=\text{9}0\text{ g peat}+\text{2}.\text{5 g perlite}+\text{7}.\text{5 g micro-zeolite};\\ \text{B2}=\text{9}0\text{ g peat}+\text{2}.\text{5 g perlite}+\text{6}.\text{5 g nanozeolite};\text{ and}\\ \text{B3}=\text{9}0\text{ g peat}+\text{2}.\text{5 g perlite}+\text{7}.\text{5 g micro-zeolite}+0.\text{2 g }{\text{CaCO}}_{\text{3}}. \end{array}$$

The formulation B1 was proven in research practice as a good carrier for alfalfa rhizobia (personal experience). The B2 and B3 are novel combinations evaluated in this study. Both types of zeolites were commercially provided: Zeolite Nanoparticles, High Purity Nano Clay Nanopowder, Nanoshel, APS <80 nm, NS6130-09-905 Purity >99% CAS No. 1318-02-1, pH 8–10; and Micronized zeolite powder particle size 0–200 µm (average 100 µm), 90%–95% clinoptilolite, pH 10.

The perlite was used to increase the porosity of inoculant and was purchased as a commercial product (Perlite P-10), with SiO_2_ 68%–75%, pH 6.0–9.0. The peat supplemented with other materials was packed into polyethylene bags, 40–50 µm thick, and then sterilized with 25 kGy gamma radiation. The ratio of C/N in final inoculants for B1, B2, and B3 was 20/1, 21/1, and 20/1, respectively. The investigation included 5 strains of *Ensifer meliloti* (218, 225, 252, 4148ss, and 4193cs) all part of the Institute of Soil Science collection (Figure S1). All five strains used in this study were previously genetically characterized [40],[41].

First, *E. meliloti* strains were grown in Erlenmeyer flasks in 20 mL yeast mannitol broth (YMB) [9] inoculated with one rhizobial colony from YMA plates on a rotary shaker (150 rpm) at 28 °C for 48 h to obtain exponential growth of rhizobia (~10^9^ cells/mL, and OD600 = 1.00). Furthermore, the inoculum broths of the *E. meliloti* strains tested were obtained in 500 mL of sterile YMB by adding 20 mL of initial cultures and incubating further at 28 °C, aerated on shaker at 150 rpm for 72 h (~10^9^ cells/mL, and OD600 = 1.00). The inoculation of polyethylene bags containing sterile carriers at a ratio of 50 mL of inoculant per 100 g of carrier was done aseptically so as to provide the ready-to-use inoculum. The initial moisture contents for B1, B2, and B3 were 55.76%, 55.53%, and 56.06%, respectively. Rhizobial inoculants in the bags were incubated for 2 weeks at 28 °C before storage. All treatments were stored at room temperature (22 ± 2 °C) for 330 days in the dark, with a humidity between 30% and 50%. The number of viable bacterial cells after the specific period of storage was determined by dilution plating. The initial point was 30 days after 2 weeks of incubation. Rhizobial number in the solid form of inoculants was determined according to the method of decimal solution from 10^−3^ to 10^−7^ on YMA with Congo red medium with 4 replicates, in the dilution where the colony count was between 30 to 120 colonies. The number of rhizobia was expressed as CFU per gram of inoculant at fresh weight base [9],[42]. Additionally, pH values were measured in all treatments.

### 2.2. Nodulation test and nitrogen-fixing efficiency

The nodulation capability of different rhizobial inoculants was tested by planting inoculated alfalfa seeds with particular carrier formulations, axenically in test tubes containing 30 mL of nitrogen-free Jensen agar medium [9]. The seeds of *Medicago sativa* L. cultivar K-28 selected at the Institute for Fodder Crops Kruševac, Serbia, were used. Sterilized alfalfa seeds (10 g) [9] were thoroughly mixed with 0.14 g of particular carrier formulations and 0.3 mL of H_2_O, aseptically, and let to dry a bit in the dark. For each formulation, one inoculated seed was aseptically plated in one test tube with Jensen agar medium. There were 10 replication per treatment. Two groups of control plants were used: plants without inoculation (Ø) in N-free medium and plants without inoculation and with mineral N supplementation (0.05% KNO_3_) (NØ) [9].

Plants were grown for 6 weeks under uniform light with a 16/8 h photoperiod, and the number of root nodules and plant height were recorded for each treatment. The plant material was dried in an oven at 70 °C and the shoot dry weights (SDW) were measured. The N percentage in the plant material was determined by a CNS analyzer, Vario model EL III.

Additionally, a germination test with inoculated seeds was performed with Jensen medium in Petri dishes. The percentage of germinated seeds were determined after 10 days of incubation in the dark.

The data for CFU of rhizobia were transformed using a logarithmic transformation (log10) to fit a normal distribution. The effect of the carrier and strain over storage time on rhizobial survival in inoculant (CFU/g) was evaluated by repeated measures using a mixed analysis of variance (ANOVA) followed by post-hoc Tukey's honestly significant difference (HSD). Due to a significant interaction between all factors, a one-way ANOVA was used to test each strain x carrier combination at each time point. The effects of the strain, carrier, and storage time on plant parameters were evaluated by a three-way ANOVA. The one-way ANOVA was used to test for significant differences among all strain x carrier combinations, as well as non-inoculated plants (Ø) and N supplemented plants (NØ). Data were processed by the IBM SPSS, 28.0 software version (SPSS, Inc., Chicago, IL), and R, version 4.2.3 (R Core Team, 2023).

## 3. Results

### 3.1. Survival of rhizobia in different carriers

The results revealed that all *E. meliloti* strains could grow well in all peat-based formulations, and were capable of surviving during prolonged storage at room temperature (Figure 1). The initial number of rhizobia in the 30^th^ day in all formulations was above 10^9^ CFU/g, which is required in bioformulations according to the local legislation and generally accepted as good quality inoculant. Moreover, the increasing number of rhizobia was detected in most of the strains and carrier combinations until the 90^th^, day when the highest number of rhizobia was detected in most of the combinations, that is, above 10^10^ CFU/g. In the 6^th^ month of storage (180 days), the decreasing number of rhizobia was detected, but was still very high, higher than 10^9^ CFU/g, in all combinations (except for strain 252 in B1 (micro-zeolite amended)). In the 330^th^ day, combinations with 218, 4148ss and 252 still had the number above 10^8^ CFU/g. During the storage, the highest number of rhizobia was detected in B2 or B3 formulations as follows: in the 30^th^ day in 4193cs-B2 (nanozeolite), in the 60^th^ day in 225-B3 (micro-zeolite + CaCO_3_), in the 90^th^ day in 4148ss in B3, in the 180^th^ 4193cs in B3, and in the 330^th^ for 252 in B3.

The ANOVA showed that the number of rhizobia is dependent on the strain, carrier and time of storage, as well as their interactions (Table 1). Therefore, an optimal carrier should be specifically selected for each individual bacterial strain, taking desirable shelf life into account (Figure 1). Among the carriers considering the whole storage period, the best carriers were B3 micro-zeolite + CaCO_3_ amended (mean value 9.47 log CFU/g) and B2 nanozeolite amended (mean value 9.42 log CFU/g), which were significantly better than B1 micro-zeolite amended (mean value 9.24 log CFU/g). For all combinations, the most performant strains for mean CFU/g were *E. meliloti* 252 and 4148ss (9.54 and 9.52 log CFU/g, respectively), which were significantly better than the other three strains.

**Figure 1.**
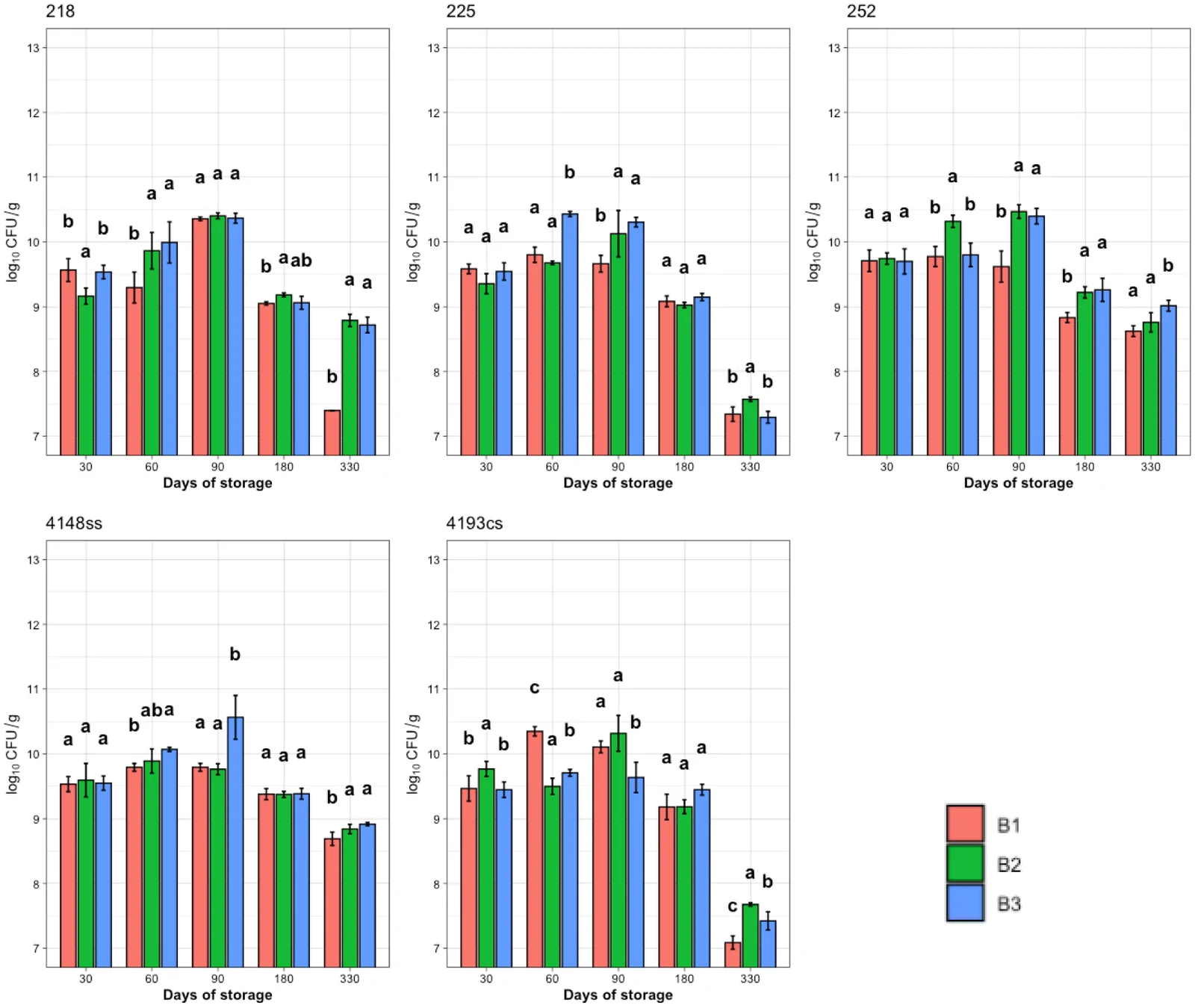
Survival of *E*. *meliloti* strains (218, 225, 252, 4148ss, and 4193cs) in different peat-based formulations during the storage. B1 (peat + perlite + micro-zeolite); B2 (peat + perlite + nanozeolite); B3 (peat + perlite + micro-zeolite + CaCO_3_). The bars represent logCFU/g (means of 4 replications) ± SD. The bars designated by the same letter are not significantly different within each time point according to Tukey's HSD test.

**Table 1.** Repeated mixed ANOVA for log_10_CFU/g of inoculant for *E. meliloti* strains in different carrier and time of storage.

Source of variation	CFU/g		
Between-subjects effects	df	*F-value*	*p-value*
Strain	4	92.818	0.000
Carrier	2	76.930	0.000
Strain x carrier	8	16.808	0.000
Within-subjects effects			
Time	4	1773.84	0.000
Time x strain	16	62.44	0.000
Time x carrier	8	14.07	0.000
Time x Strain x Carrier	32	15.74	0.000

*p* < 0.05 significant, *p* < 0.01 highly significant, *p* < 0.001 very highly significant.

### 3.2. Efficiency of peat-based carrier with zeolite amendments in alfalfa

The growth parameters of alfalfa plants inoculated with different inoculants stored for 180 and 330 days are presented in Figure 2. The nodulation was 100% when inoculated with inoculant stored for 180 as well as stored for 330 days at room temperature, that is, in all inoculated plants nodules were formed.

In plants inoculated with 180 days-stored inoculants, the parameters measured showed that the best for nodulation was the 252 strain (in B1 and B3 carriers), which formed the highest number of nodules, but not statistically significant from other numerous treatments. In addition, the highest values of the SDW were detected in strain 252 B3 and strains 4148ss and 4193cs in all carriers B1, B2, and B3. If we consider the different carriers with the same strain, there were no differences in the SDW among carriers except for strain 252 (Figure 2 and Table 2).

In the 330th day, the strains 218 (B1 and B2), 4148ss (B1 and B3), and 4193cs (B1 and B3), together for 225 (B2 and B3), were the most performant for the SDW (Figure S2). These SDW values were not significantly different from the control plants grown in the medium with optimal mineral N concentrations (NØ), indicating good N fixation capacity of the inoculants. The SDW increased in the inoculated plants from 2.3 to 5.3 times for 180 days-stored inoculants, compared to the non-inoculated plant (Ø), and 2.4 to 5.4 times in 330^th^ day stored inoculants.

The N percentage in inoculated plants ranged between 3.23% to 4.07%, from 1.45%–1.65% in non-inoculated plants (Ø), and 3.6%–3.9% in N supplemented plants (NØ). The N content in all inoculated plants was significantly higher than in non-inoculated plants for both the storage period of the inoculants and similar to NØ supplemented plants for all strain and carrier combinations (except strain 225) (Figure 2). The total N content, in mg/plants, were highly corelated to the SDW of the plants (Table 3). The plants height was highly corelated to the SDW and N content, while there was no correlation between the nodule number and other parameters (Table 3).

**Table 2.** Analysis of variance (ANOVA) of all studied parameters of alfalfa under the different rhizobial inoculant formulation.

Source of variation		SDW		Nodule number	
	df	*F-value*	*p*-value	*F-value*	*p*-value
Strain	4	8.529	0.000	1.960	0.104
Carrier	2	0.422	0.656	0.092	0.912
Storage	1	2.214	0.139	28.901	0.000
Strain x Carrier	8	2.029	0.047	0.914	0.506
Strain x Storage	4	9.244	0.000	2.854	0.026
Storage x Carrier	2	0.176	0.839	0.867	0.422
Strain x Carrier x Storage	8	0.618	0.762	0.759	0.640
Source of variation		Plant height		Nitrogen content	
	df	*F-value*	*p*-value	*F-value*	*p*-value
Strain	4	11.047	0.000	8.938	0.000
Carrier	2	2.463	0.089	0.441	0.644
Storage	1	4.623	0.033	3.096	0.081
Strain x Carrier	8	1.288	0.254	2.706	0.008
Strain x Storage	4	8.584	0.000	9.888	0.000
Storage x Carrier	2	2.832	0.062	0.201	0.818
Strain x Carrier x Storage	8	2.571	0.012	1.092	0.372

SDW—shoot dry weight; *p* < 0.05 significant, *p* < 0.01 highly significant, *p* < 0.001 very highly significant.

**Figure 2.**
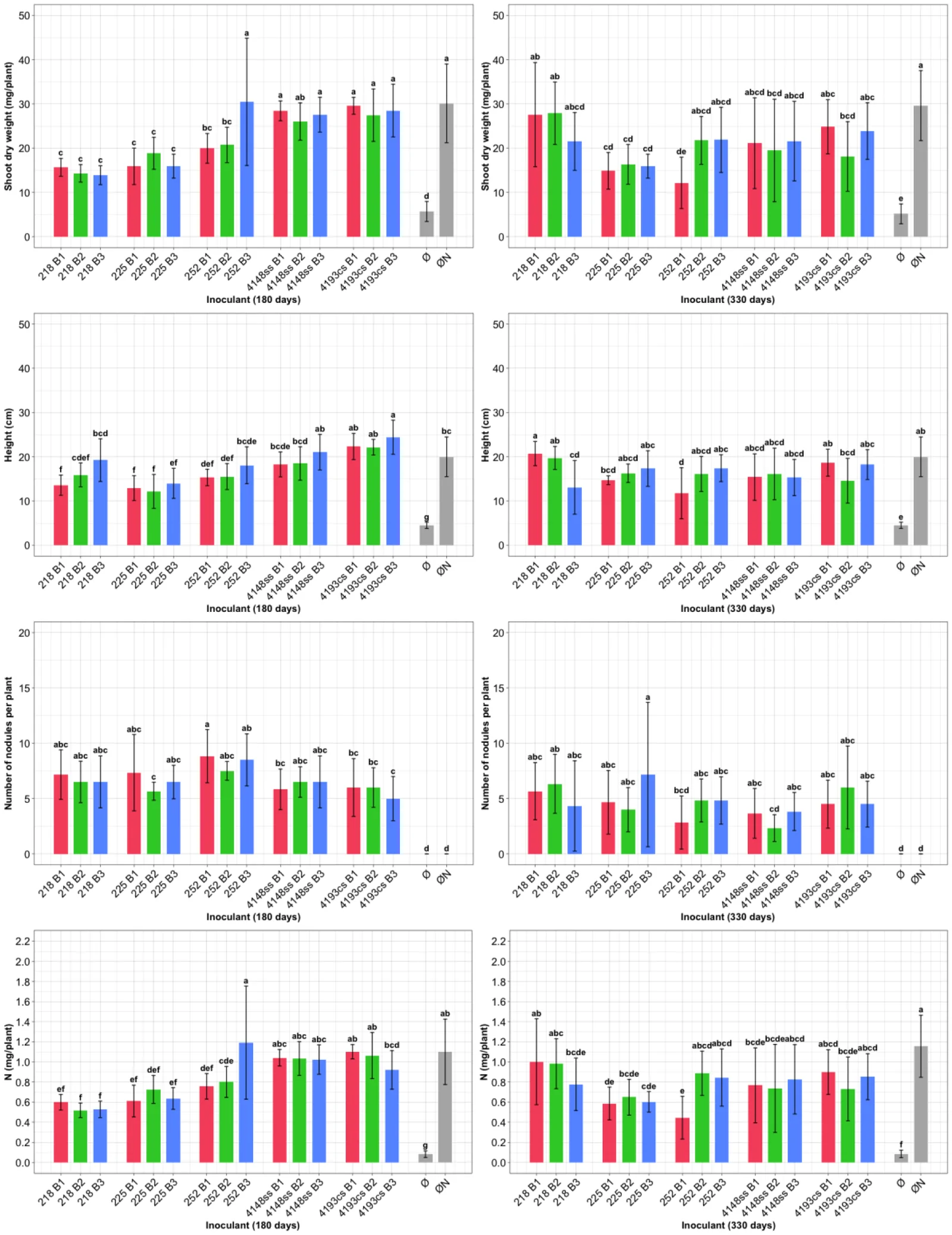
Plant growth parameters of alfalfa under the different rhizobial inoculant formulations stored for 180 and 330 days. *E. meliloti* strains (218, 225, 252, 4148ss, and 4193cs). Ø Non-inoculated control (no inoculation and no N supplementation). NØ Treatment with N supplementation and without inoculation. B1 (peat + perlite + micro-zeolite); B2 (peat + perlite + nanozeolite); B3 (peat + perlite + micro-zeolite + CaCO_3_). The bars represent means of 6 replications ± SD. The bars designated with the same letter are not significantly different according to Tukey's HSD test.

**Table 3.** Correlation coefficients between alfalfa growth parameters.

	Height 180	Nodule 180	SDW 180	Nitrogen 180	Height 330	Nodule 330	SDW 330	Nitrogen 330
Height 180	1							
Nodule180	−0.435	1						
SDW 180	0.719**	−0.188	1					
Nitrogen 180	0.630*	−0.083	0.979**	1				
Height 330	−0.011	−0.279	0.015	−0.030	1			
Nodule 330	−0.154	−0.115	−0.358	−0.358	0.497	1		
SDW 330	0.331	−0.285	0.100	0.029	0.761**	0.279	1	
Nitrogen 330	0.314	−0.254	0.147	0.097	0.749**	0.298	0.977**	1

*significant at the 0.05; **significant at the 0.01 probability level.

The pH of the peat used in this study was 7.50 and the additives additionally increased the pH of the carrier formulations (B1, B2, B3: pH 8.06, 7.99, and 8.03, respectively) (Figure 3), still being suitable for rhizobia growth, since *Ensifer* as a fast-growing bacterium tolerates between 5–10.5 pH.

**Figure 3.**
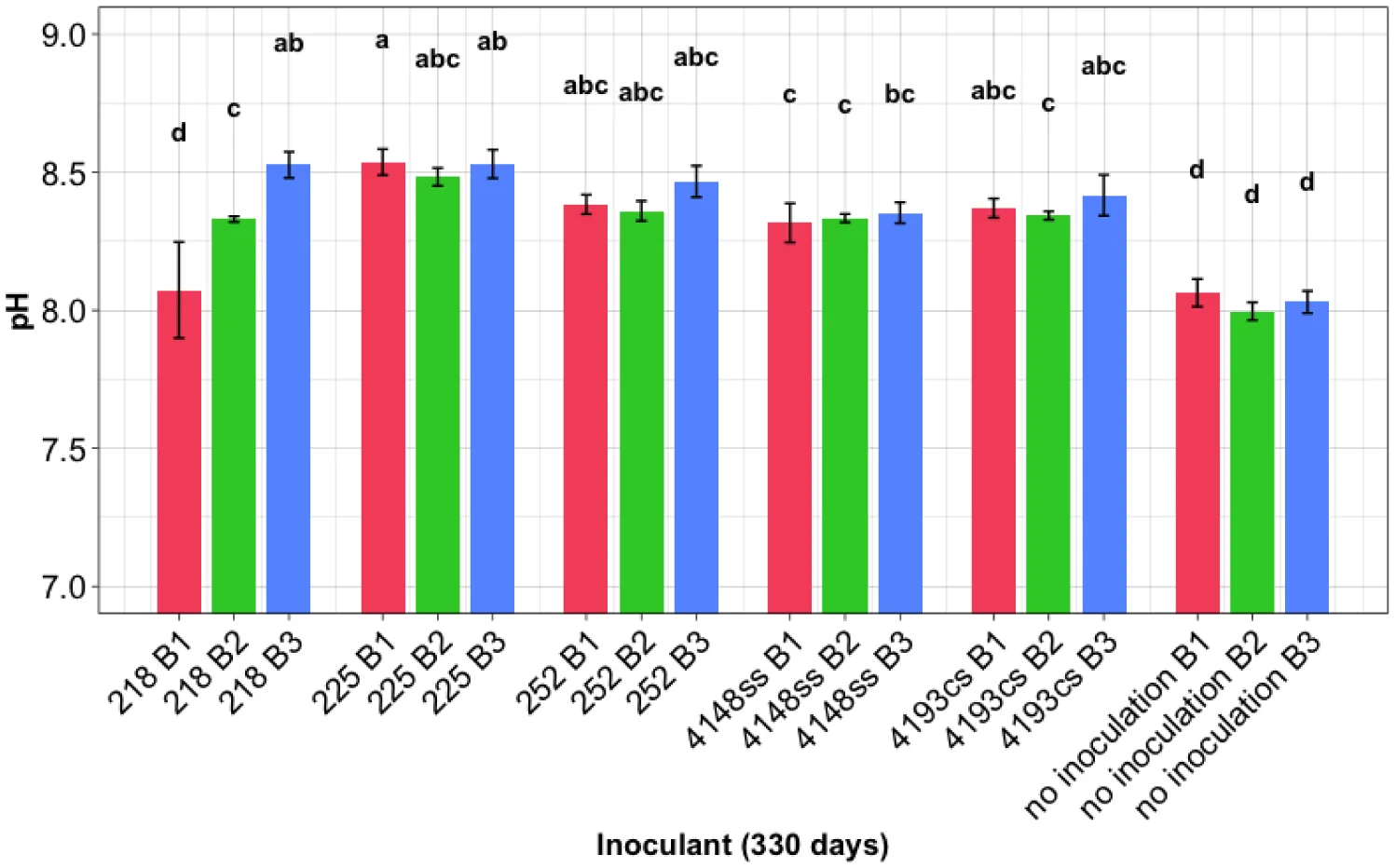
Values of pH in different peat-based carrier formulations after 330 days of storage. *E. meliloti* strains (218, 225, 252, 4148ss, and 4193cs). B1 (peat + perlite + micro-zeolite); B2 (peat + perlite + nanozeolite); B3 (peat + perlite + micro-zeolite + CaCO_3_). The bars represent pH (means of 3 replications) ± SD. The bars designated by the same letter are not significantly different according to Tukey's HSD test.

## 4. Discussion

### 4.1. Effect of different carriers on rhizobial inoculant survival

During the decades, peat has been proven as a proper carrier for rhizobial inoculants for different leguminous species [21],[39],[43]. Additionally, perlite was applied as either a sole carrier or in the mixture with peat as well as other substrates to improve the aeration and to balance the water retention capacity of the inoculant [13],[39],[44]. Perlite has a much lower water holding capacity than most organic materials and is often added to soilless media to increase the drainage and porosity [45]. Zeolite, with its high cation-exchange capacity and porous structure, enhances nutrient retention and supports PGPR colonization [21]. In addition, calcium salts were used in seed priming and peat inoculants [33]–[36]. Recently, special attention was focused on the nanomaterials as a carrier, particularly nano-zeolites as a promising material in development of better soil conditioners, growth medium in nurseries, and nano-fertilizers [46], but their scalability remains limited [21].

In this research, the combination of peat and previously mentioned additives was tested as carrier for alfalfa rhizobia. The results of our research showed that the mixture of peat and perlite, supplemented with micro-zeolite+CaCO_3_ (B3) and nanozeolite (B2), had better potential for preservation of rhizobia than micro-zeolite alone (B1), considering the hole storage period of 11 months.

The decreasing number of CFU was detected in 6^th^ month of storage and decreased further in the 11^th^ month. In the 6^th^ month of storage, the CFU in all combinations (except one) exceeded 10^9^ per gram of inoculant, which is considered as high-quality inoculum [11],[43],[47]. Peat-based inoculant supported cell survival of above 10^7^ cells/g for fast-growing rhizobial strains after six months of storage [34]. Moreover, the survival of the rhizobia was higher in the solid carriers, particularly peat-based inoculant than in the liquid carriers, including strain of *Sinorhizobium* surviving better for 219% compared to other carrier formulations [47].

The good effects of zeolite as a carrier material are recognized. Zeolites act as strong cation exchangers: they are effective carriers of herbicides, fungicides, and pesticides, and capture cationic contaminants in environmental cleaning processes [30],[48],[49]. In combination with fertilizers, zeolites may help to buffer the soil pH levels [29],[50]. Zeolite characterized as natural absorbent was used in N-fertilizers with the aim of controlled release of nutrients [30].

In our study, better performances of the B2 carrier with nanozeolite as an additive was detected. Nanoparticles have a higher surface tension, which influences their potential to absorb and slow release the nutrients and moisture to and from the environment. They have a higher reactivity in comparison to the particle size (micron or millimeter size) present in bulk [22],[23]. The application of nano-zeolite had a positive impact on the bacterial growth and protein expression [24]. Additionally, nano-zeolite also enhanced protein expression and supported the growth of PGPR in nutrient broth [24]. It is considered that nanozeolite can be used to support the growth of PGPR for a longer time due to the slow release of nutrients and offers an environmentally sustainable approach to increase crop production, which is easily degradable and does not affect the microbial activity in the soil [24],[26].

In our study, rhizobia survived better in the presence of micro-zeolite and CaCO_3_ than micro-zeolite alone. The pH of the medium or CaCO_3_ structure can be responsible for the survival of rhizobia in carrier with CaCO_3_. Calcium salts were often used in seed priming and peat inoculants as well as for the pH adjustment of different basic carriers [33],[34],[36],[51]. The pH is the factor that commonly affects the survival of rhizobial strains in carriers [47]. *Sinorhizobium* (*Ensifer*) is a fast-growing rhizobium and usually tolerates between 5–10.5 pH [42], which is also confirmed for our tested strains [40]. Adding CaCO_3_ provides a stable, residual buffer that maintains a neutral-to-alkaline pH over extended periods. In general, fast-growing rhizobia strains produce acids when grown on sugars (such as yeast extract-mannitol media), while growth on organic acids or amino acids results in alkalinization, but this can vary depending on the strain and the composition of the medium [9],[52],[53]. This can be the case for the further increase in pH over time, rather than decrease. Additionally, the change in pH depends upon organic matter and clay content as well as the buffering capacity of the carrier [42].

Although nanozeolites are generally considered safe, they are still undergoing testing due to the toxicity of some nanomaterials [46]. In the short-term study, four sizes, namely 10–100 nm, 200–400 nm, 500–1000 nm and 1–2 µm, at 100, 1000, and 2000 ppm were tested, and all zeolites positively influenced the growth dynamics of all four bacterial genera tested, including *Rhizobium* [46]. In the same study, a lactose dehydrogenase assay showed that 2000 ppm nanozeolite exhibited cytotoxic effects on the microorganisms tested, while a comet assay demonstrated no quantifiable DNA damage in the nanozeolite-treated cells, thus indicating the necessity for further nanozeolite toxicity testing [46]. In our research, much higher concentrations (about 6%) of nanozeolite apparently did not show negative effects on rhizobial survival nor for N efficiency of rhizobial strains almost for almost one year of storage.

In our study, the rhizobia inoculants were kept at room temperature, which is the most common way for practical storage of inoculants. Generally, the growth and survival of different rhizobia in peat and perlite-based inoculants showed similar trends for all strains in both carriers, but better survival was observed at 4 °C compared to 28 °C [54]. The survivals of rhizobial strains on zeolites kept at 25 °C for 90 days indicated a capacity of zeolite to maintain an adequate survival rate for the rhizobial strains, although, during the time, the number of rhizobia decreased [25]. In another study, after 240 days at 25 °C, the survival of the rhizobia (*Sinorhizobum*) remained at 2 × 10^9^ cells/g, which is the suggested number for biofertilizers [47].

### 4.2. Plant growth and nodulation of alfalfa inoculated with different inoculant after storage

Besides providing the increased microbial number and prolonged survival, the effectiveness of inoculant should be retained during storage [55],[56]. However, despite nanomaterial potential as carriers for PGPR, their efficiency still remains underexplored [21]. Some research indicates the efficiency of combined treatment of nanozeolite and bioinoculants for promoted maize plant growth [57], and a higher efficiency of nano inoculant over regular ones after six months of storage [37]. Nano-clay (Montmorillonite) and NCNPs (natural char nanoparticles) were the best carriers for *Pseudomonas* strains (phosphate solubilizing bacteria) in the alginate beads, thereby maintaining viability and efficacy in phosphate solubilization after six months of storage at both tested temperatures of 4 °C and 28 °C [37]. Nanogypsum (50 mg/L) also supported the growth and protein contents of plant growth-promoting *Pseudomonas taiwanensis* and *Pantoea agglomerans* in liquid media [57]. Subsequently, the inoculants are expected to have good nodulation properties during the first six months of storage period, and we tested their effectiveness at this point and after 11 months of storage.

In our research, all strains kept on the different carriers significantly increased alfalfa growth after 180 and 330 days of inoculant storage, thereby providing SDW up to 5 times higher compared to control non-inoculated plants. The SDW depended on the strain applied (Table 2), being the highest for 4193cs and 4148ss (both storage time). In contrast to better survival of strains in B2 and B3 carrier formulations, the influence of the carrier was not detected in alfalfa plants testing. We assume that the sufficiently high number of rhizobia for symbiotic effectiveness was present in all formulations even after 330 days of storage. Moreover, all combinations of rhizobial strains and carriers effectively nodulated alfalfa plants after 180 and 330 days of inoculant storage. The nodule number did not depend on the strain and the carrier formulation only on the time (being lower in 330^th^ day). Consequently, no correlation (Table 3) was detected between nodule formation in the host plant and symbiotic capacity. The N percentage, and consequently the N content (mg/plant) were significantly higher in inoculated plants, which also confirmed the efficiency of inoculants. The peat-based inoculants were confirmed for rhizobial efficiency preservation during the storage for soybean, common bean rhizobia, being tested in the controlled and field conditions [47],[56]. It was noted that for the *Sinorhizobium fredii* strain, the most promising carrier was peat, thus providing the best survival for this rhizobium after prolonged storage of 7 months [34].

### 4.3. Study limitations and future research

However, according to our previous research experience, it was expected that perlite and zeolite improve peat carriers, and one of the limitations of our study was the absence of a peat-only control (without any other additives). In addition, the testing of biosafety of nanozeolite was not included in the study. All experiments with plants in this research were performed under axenic plant testing, so further research of carrier formulations under semi-controlled and the field conditions would provide more data regarding the efficiency of nanozeolite and other formulations. It might even be expected to achieve better results in the field, under environmental conditions, where numerous factors can affect the inoculant efficiency, so the protective effect of the additive could be more pronounced.

## 5. Conclusions

The results of the study demonstrated that the carriers B3, combination of peat + perlite amended with micro-zeolite + CaCO_3_, and B2 amended with nanozeolite, exhibited superior characteristics over peat + perlite and micro-zeolite in prolonged storage under room temperature for all tested *E. meliloti* strains. The developed carrier formulations exceeded >10^9^ CFU/g after six months of storage, and >10^8^ CFU/g after eleven months of storage. The symbiotic efficiency of inoculant formulations with alfalfa was high after 180 and 330 days of inoculant storage and depended on the strain. This research combined highly efficient rhizobial strains in an optimized carrier for inoculant development for prolonged storage up to one year, and further confirmation of results should be done under environmental conditions.

## Use of AI tools declaration

The authors declare they have not used Artificial Intelligence (AI) tools in the creation of this article.


