# Supplementary material for: Zeolite-amended peat carriers improve long-term survival of Ensifer meliloti and maintain alfalfa nodulation efficiency
Source: AIMS Microbiol. 2026 Sep 15;12(3):666–81. doi: 10.3934/microbiol.2026029 (PMC13652751; doi:10.3934/microbiol.2026029)
Supplement: Supplementary file 1 [file microbiol-12-03-029-s001.pdf]

AIMS Microbiology

AIMS Microbiology, 12(3): 666–681.
DOI: 10.3934/microbiol.2026029
Received: 05 April 2026
Revised: 26 August 2026
Accepted: 07 September 2026
Published: 15 September 2026

https://www.aimspress.com/journal/microbiology

Research article

Zeolite-amended peat carriers improve long-term survival of *Ensifer meliloti* and maintain alfalfa nodulation efficiency

Olivera Stajković-Srbinović[1,*], Mila Pešić[1], Biljana Sikirić[1], Vesna Mrvić[1], Snežana Andjelković[2] and Dušica Delić[1]

[1] Institute of Soil Science, Teodora Drajzera 7, 11000 Belgrade, Serbia
[2] Institute for Forage Crops Kruševac, Globoder, Kruševac, Serbia

* **Correspondence:** Email: oliverastajkovic@yahoo.com; Tel: +381698223532; Fax: +381112667123.

Supplementary

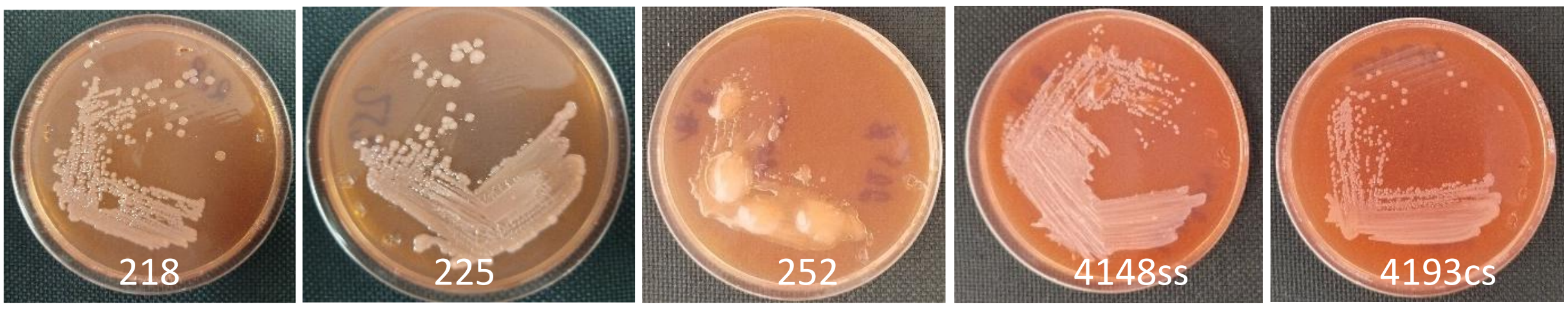

Figure S1. *E. meliloti* strains (218, 225, 252, 4148ss, 4193cs) grown on Yeast Mannitol Agar plates with Congo Red.

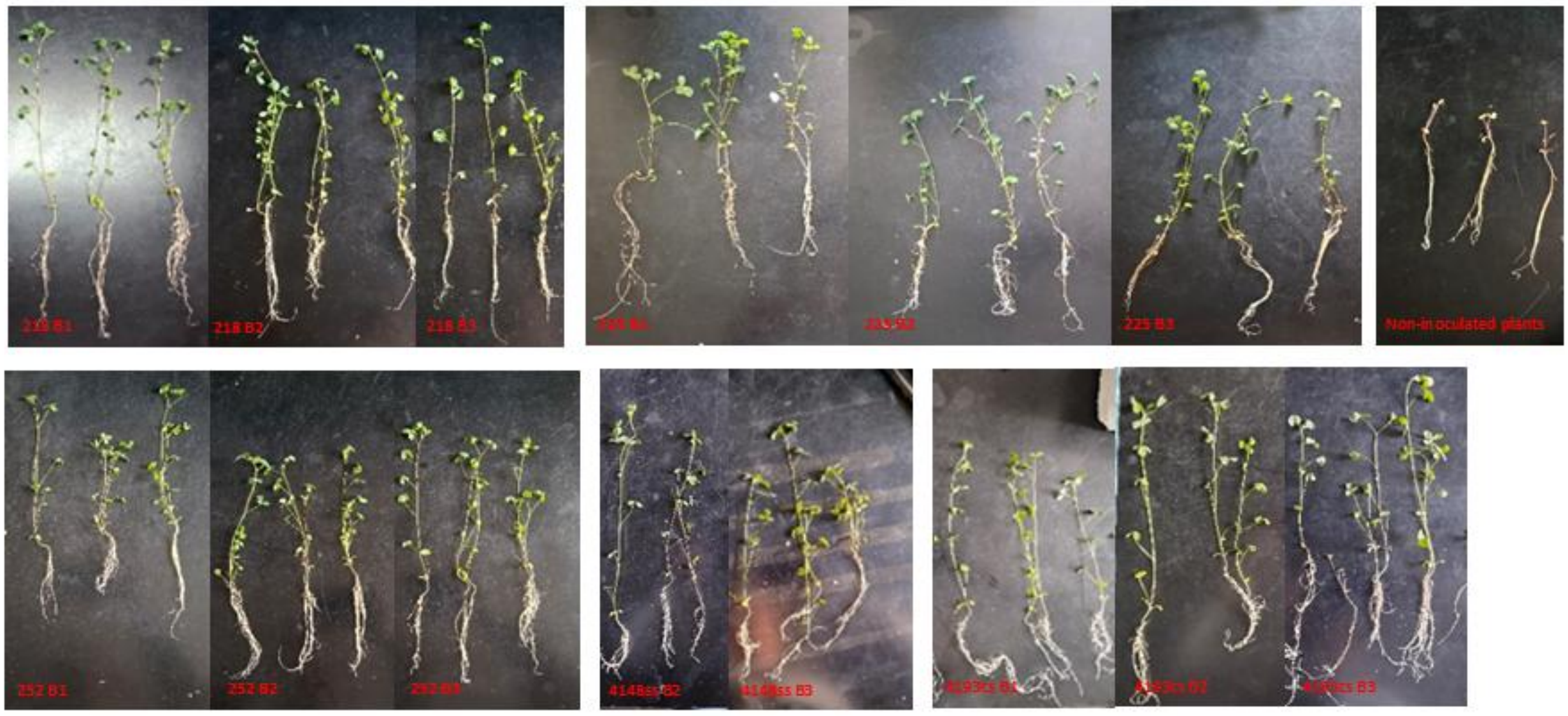

Figure S2. Plants inoculated with different strain and carrier formulations sored for 330 days. *E. meliloti* strains (218, 225, 252, 4148ss, 4193cs). Different peat-based formulations during the storage. B1 (peat + perlite + micro-zeolite); B2 (peat + perlite + nanozeolite); B3 (peat + perlite + micro-zeolite + $CaCO_3$). Ø Non-inoculated control (no inoculation and no N supplementation).

AIMS Press

© 2026 the Author(s), licensee AIMS Press. This is an open access article distributed under the terms of the Creative Commons Attribution License (https://creativecommons.org/licenses/by/4.0)
